# Supplementary material for: Whole-body PET imaging of simian immunodeficiency virus using gp120-targeting probes fails to reveal regions of specific uptake in rhesus macaques
Source: Eur J Nucl Med Mol Imaging. 2025 Jan 31;52(7):2645–57. doi: 10.1007/s00259-025-07110-8 (PMC12119729; doi:10.1007/s00259-025-07110-8)
Supplement: Supplementary file 1 — Supplementary Material 1 [file 259_2025_7110_MOESM1_ESM.docx]

**Whole-body PET imaging of simian immunodeficiency virus using gp120-targeting probes fails to reveal regions of specific uptake in rhesus macaques**

**Sharat Srinivasula^1^, Insook Kim^2^, Hyukjin Jang^1^, Paula Degrange^3^, Heather Brown^3^, Viviana Dalton^3^, Yunden Badralmaa^4^, Ven Natarajan^4^, Brad Long^3^, Jorge A. Carrasquillo^5^, and Michele Di Mascio^6*^**

1. AIDS Imaging Research Section, Clinical Monitoring Research Program Directorate, Frederick National Laboratory for Cancer Research, Frederick, Maryland, USA
2. AIDS Imaging Research Section, Applied/Developmental Research Directorate, Frederick National Laboratory for Cancer Research, Frederick, Maryland, USA
3. AIDS Imaging Research Section, Laulima Government Solutions, Integrated Research Facility, NIAID, NIH, Frederick, Maryland, USA
4. Laboratory of Molecular Cell Biology, Frederick National Laboratory for Cancer Research, Frederick, Maryland, USA
5. Molecular Imaging Program, Center for Cancer Research, NCI, NIH, Bethesda, Maryland, USA
6. AIDS Imaging Research Section, Division of Clinical Research, NIAID, NIH, Poolesville, Maryland, USA

**Corresponding author:** Dr. Michele Di Mascio ([mdimascio@niaid.nih.gov](mailto:mdimascio@niaid.nih.gov))

**Materials and Methods**

**Animals**

All animal procedures were performed at the National Institutes of Health (NIH) Animal Center. Animals’ health was monitored daily and are cared for according to the *Guide for the Care and Use of Laboratory Animals*, 8^th^ Ed., Animal Welfare Act regulations, and policies of NIH, an AAALAC-accredited facility. Macaques were individually housed in 18 cu ft stainless steel cages on a 12-hour light/dark cycle in a temperature-controlled indoor facility with access to behavioral enrichment toys and were fed Purina Old World Primate Diet with rotating enrichment food. Animals were anesthetized with standard doses of Ketamine or Telazol for procedures considered to cause pain or distress to humans.

**Antiretroviral therapy**

The daily antiretroviral therapy (ART) regimen consisted of three antiretroviral drugs comprising two reverse transcriptase inhibitors and one integrase inhibitor: Tenofovir (PMPA, 20mg/kg, Gilead) and Emtricitabine (FTC, 30mg/kg, Gilead), each injected subcutaneously once a day in the scapular area of the back, and either Raltegravir (20mg/kg, Merck) given twice a day mixed in food, or L-870812 (20mg/kg (max 100mg), Merck) given once a day mixed in food.

**Preparation of F(ab’)_2_, conjugation of p-SCN-Df, and ^89^Zr radiolabeling of anti-gp120 mAbs**

*F(ab’)_2_ preparation:* We prepared ITS103.01LS-F(ab’)_2_ by pepsin digestion according to the manufacturer’s instruction (Pierce F(ab’)_2_ Preparation Kit, Thermo Fisher Scientific). Briefly, 20 mg of ITS103.01LS was digested by pepsin immobilized on agarose gel (Thermo Fisher Scientific) in 0.1M sodium acetate buffer (pH 4.4). The digestion was performed at 37°C for 1h using an optimal antibody/pepsin weight ratio between 3.5-5:1. After digestion, the reaction mixture was purified using Protein-A Sepharose affinity chromatography (Thermo Fisher Scientific), followed by dialysis in PBS at 4°C for 22h, using a dialysis membrane cassette with 20 kDa molecular weight cut-off. The purified protein was concentrated using an Amicon Ultra (MilliporeSigma) to 13 mg/mL. Protein purity was assessed by SDS-PAGE (Invitrogen) and size-exclusion HPLC (Gilson, Middleton, WI) equipped with a size-exclusion TSK gel G3000SWxL column (7.8 × 300 mm, 5 μm, TOSOH Bioscience; 0.067M sodium phosphate/0.15M sodium chloride with 0.1M KCl, pH 6.8; 1.0 ml/min) and a UV monitor.

*Conjugation of p-SCN-Df to mAbs:* The bifunctional chelating agent p-isothiocyanatobenzyl-desferrioxamine (Df-Bz-NCS) was conjugated with lysine residues of the mAbs following Vosjan et al [1]. Briefly, for *in-vitro* assays, 200-250µg of each anti-gp120 mAb was reacted with Df-Bz-NCS at a molar ratio of 1:5 for 7D3 (1:3 for ITS103.01LS-F(ab’)_2_) in 0.1M sodium bicarbonate, pH 9.5, 37°C for 1 h. For *in-vivo* PET imaging, 4mg of intact 7D3 or ITS103.01LS-F(ab′)_2_ mAb was used for conjugation each time. The Df-conjugated mAbs were purified with a Zeba Spin Desalting column (7K molecular weight cutoff (MWCO), Pierce Biotechnology, Rockford, Illinois) pretreated with 0.25M sodium acetate buffer.

*Radiolabeling:* [^89^Zr]Zr-Df-7D3 and [^89^Zr]Zr-Df-ITS103.01LS-F(ab′)_2_ were prepared as previously described but with minor modifications [1]. Briefly, 37 MBq of ^89^Zr (or 370 MBq for *in-vivo*) in 1M oxalic acid was neutralized with 2M sodium bicarbonate, followed by the addition of 0.5M HEPES buffer (pH 7.1-7.3) and gentisic acid (25mg/mL, 5µL, pH 6.5). Then, 200µg of Df-conjugated mAb for *in-vitro* and 3.5 mg for *in-vivo* studies was added, and the pH was adjusted to 6.8-7.2, incubating for 1h at 30°C. The labeled product was purified by eluting with PBS 1X (pH 7.2) through a PD-10 column pretreated with 25mg BSA containing 1 µmol DTPA to block nonspecific protein binding sites. The radiochemical purity was assessed by analytical SE-HPLC (Gilson, Middleton, WI) using a column (TSK G4000PWxL (7.8×300 mm, 10μm, TOSOH Bioscience; 0.067M PBS with 0.1M KCl, pH 6.8; 1.0 mL/min) equipped with an online flow radioactivity detector (Bioscan Inc., Washington, DC). The radiolabeling yield was determined based on the distribution of ^89^Zr activities between ^89^Zr labeled mAb and unbound ^89^Zr on iTLC and/or HPLC profiles obtained before the purification. The radiochemical purity utilized in all studies was 100%.

**Binding specificity assays of radiolabeled anti-gp120 mAbs**

Cells were washed and 1-8 million viable cells per well were suspended in 100µL (for non-specific binding) or 125µL (for total binding) of media. To measure total binding (TB), cells were incubated with 50µL of [^89^Zr]Zr-7D3 (incubation concentration 0.15nM-6.7nM) or [^89^Zr]Zr-ITS103.01LS-F(ab’)_2_ (incubation concentration 0.5nM) for 90 min at 4^o^C on a rocker. To measure non-specific binding (NSB), cells were preincubated with 25µL of unlabeled 7D3 or ITS103.01LS-F(ab’)_2_ (incubation concentration 1000nM) for 20 min at 37^o^C in a humidified 5% CO_2_ incubator, and subsequently with 50µL of [^89^Zr]Zr-7D3 (incubation concentration 0.15nM-6.7nM) or [^89^Zr]Zr-ITS103.01LS-F(ab’)_2_ (incubation concentration 0.5nM) for 90 min at 4^o^C on a rocker. After the incubation, samples were microcentrifuged at 12,000g for 5 min (Eppendorf 5415C), supernatant aspirated and discarded, and the counts per minute (CPM) in the cell pellets were measured in a gamma counter (PerkinElmer 1480 Wizard or 2480 Wizard^2^). The media used is as follows: RPMI-1640 without L-Glutamine, 10% Heat Inactivated FBS, and supplemented with Penicillin-Streptomycin for SIV1C cells; RPMI-1640 without L-Glutamine, 10% Heat Inactivated FBS, 1% L-Glutamine, 1% Hepes Buffer, and supplemented with Gentamicin for MT4 cells; PBS (phosphate buffered saline) pH 7.4 for PBMC, LNMC, and spleen cells.

In one assay where the NSB wells were missing due to an insufficient amount of unlabeled mAb, the TB of uninfected MT4 cells was substituted for NSB to estimate the binding specificity of the radioligand to SIV Env expressing cells.

For the secondary binding specificity assay, immuno-breakable well strips (Thermo Scientific) were coated with 150µl of SIV gp120 (4.0 µg/ml in phosphate-buffered saline (PBS), pH 7.2), incubated overnight at 4°C, and then washed with 0.05% Tween-20 in PBS (Washing buffer, Sigma Aldrich). The wells were then preincubated with either 200µl of BSA blocking buffer (Thermo Scientific; 5% bovine serum albumin in PBS) for total binding (TB) or 200µl of unlabeled 7D3 or ITS103.01LS-F(ab’)_2_ (1000nM in BSA blocking buffer) for non-specific binding (NSB) for 1h at 37°C. The wells were then washed and incubated at various concentrations [0.05nM-50nM] of 150µl of [^89^Zr]Zr-7D3 or [^89^Zr]Zr-ITS103.01LS-F(ab’)_2_ for an additional 1h at 37°C. After the incubation, the wells were washed, the dried breakable wells were detached individually, and the radioactivity (CPM) in each well was measured using a gamma counter (PerkinElmer 2480 Wizard^2^). The wells were washed thrice with 200µl of the Washing buffer during each washing step.

**Endogenous proteins competing for the anti-gp120 mAb binding site and immunogenicity of murine 7D3 or rhesus ITS103.01LS-F(ab’)_2_**

Monkey plasma was incubated with [^89^Zr]Zr-7D3 or [^89^Zr]Zr-ITS103.01LS-F(ab’)_2_ (mAb concentration 1.5nM-5nM) for 30 min at 37°C in a humidified 5% CO_2_ incubator. After incubation, a 50µl aliquot of the incubated mixture was run through size-exclusion HPLC, and a 20µl aliquot of the incubated mixture was added to 1-2 million viable gp120 expressing cells in 180µL. After 90 min incubation (mAb concentration 0.15nM-0.5nM) on a rocker at 4°C, the total incubated radioactivity CPM was measured in a gamma counter (PerkinElmer 1480 Wizard or 2480 Wizard^2^). The cell mixture was then microcentrifuged at 12,000g for 5 min (Eppendorf 5415C), supernatant aspirated and discarded, the CPM in the cell pellet measured, and the percent of total incubated radioactivity bound to gp120 expressing cells was determined.

***In-vitro* autoradiography**

20µm thick slices of axillary and inguinal lymph nodes, spleen, colon, and jejunum tissues were sectioned with a cryostat (Leica Biosystems CM1900 UV), mounted on silanized slides, dried at room temperature for 1-2h, and stored at -80^o^C when not immediately used. The fresh slides were dried for 1-2h whereas slides stored at -80°C were air-dried overnight at room temperature before use. The dried slides were pre-incubated in the buffer for 15 min at room temperature. To measure total binding (TB), slides were incubated with 1nM of [^89^Zr]Zr-7D3 or [^89^Zr]Zr-ITS103.01LS-F(ab’)_2_ for 90 min at 4^o^C. To determine non-specific binding (NSB), the slides were preincubated with 100nM of unlabeled 7D3 or ITS103.01LS-F(ab’)_2_ for 20 min at room temperature and subsequently incubated with 1nM of [^89^Zr]Zr-7D3 or [^89^Zr]Zr-ITS103.01LS-F(ab’)_2_ for 90 min at 4^o^C. After incubation, slides were rinsed twice (5 min per rinse) in cold buffer and air-dried for 1h. All incubation and rinsing steps were performed with 50mM Tris-HCL buffer (pH 7.4). The dried slides were exposed to a storage phosphor screen along with [^89^Zr]Zr-7D3 or [^89^Zr]Zr-ITS103.01LS-F(ab’)_2_ standards. After 20-24h of exposure, the phosphor screen was read using a phosphorimager (GE Healthcare Typhoon FLA 7000) at 25µm pixel resolution. Regions of interest (ROIs) were drawn manually on the images and the average intensity was extracted from each tissue using image analysis software (GE Healthcare ImageQuant TL). The binding specificity is calculated as the average intensity ratio of TB to NSB.

**PET/CT imaging and data analysis**

Animals were initially anesthetized with a restraint dose of ketamine (10 mg/kg, intramuscular) and after shaving the skin and prepping the insertion site, a 22-gauge catheter was inserted in the saphenous vein of the leg for bolus injection of the radiotracer or continuous administration of anesthetics (propofol 0.2 mg/kg/min infusion) during the imaging procedure. The macaque’s arms and legs were restrained and positioned in supine orientation for imaging. Anesthetized primates were monitored with a pulse oximeter and thermometer, and the body temperature during imaging was maintained with the Bair Hugger patient warming system.

Whole-body CT acquisitions were performed using the following parameters: Semi-circular multi field-of-view (FOV), 360 projections, 80kVp, 710µA, 90ms exposure time per projection, and 1:4 binning. Immediately following the CT scan, animals underwent a whole-body static PET scan from the top of the head to mid-thighs at one or multiple time points post radioligand injection (Table S1 and S2). PET static scans were acquired at 10 min per FOV, and 6-bed positions were scanned with a 35% overlap between the FOVs with 1-9 coincidence mode and a 5 ns coincidence time window. Raw CT data was reconstructed with scatter correction. Raw PET data was reconstructed using the following parameters: 400-600 keV energy window, 1-9 coincidence mode, Tera-Tomo 3D reconstruction with median and spike filter on, voxel size 1 mm, and 8 iterations and 9 subsets. Reconstructed PET images were corrected for attenuation (using CT material map segmentation), radioactive decay, uniformity, random coincidences, scattering of radiation, and the decay reference was set to radioligand administration time. A Gaussian post-processing filter with kernel size = 3 and sigma = 0.8 was applied to smooth the reconstructed PET image. Final CT and PET images were saved in DICOM format.

Whole blood and plasma SUV were measured by drawing blood and counting 50µL or 100µL aliquots in a gamma counter. CPM/mL was then converted to SUV correcting for radioactive decay and adjusting for counting efficiency, body weight, and injected radioactivity. Whole blood and plasma SUV were calculated at each imaging timepoint and additionally at ~13h post-injection (for [^89^Zr]Zr-7D3) or ~9h and ~24h post-injection (for [^89^Zr]Zr-ITS103.01LS-F(ab’)_2_).

**PET SUV normalization**

To control for differences in the clearance of the radioligand from the intravascular compartment between the animals, blood-adjusted tissue uptake (rSUVmax and rSUVmean) was calculated by normalizing the tissue uptake on blood SUV obtained from heart blood pool VOI ie SUVmax or mean of tissue/SUVmean of the blood pool. The heart blood pool SUVmean extracted from the 48 PET images correlated strongly with whole-blood and plasma SUV calculated from counting aliquots in a gamma counter (ρ ≥0.92, P <0.0001). Hence, the cardiac blood pool SUVmean from the PET image was used for normalization.

**Ex-vivo analysis**

One chronically SIV-infected RM and one uninfected RM pair at ~44h post [^89^Zr]Zr-7D3 injection, another pair on Day 7 post [^89^Zr]Zr-7D3 injection, and another pair on Day 5 post [^89^Zr]Zr-ITS103.01LS-F(ab’)_2_ injection were euthanized and individual LNs (axillary, inguinal, submandibular, mesenteric, and retroperitoneal), and small aliquots of the spleen and gut sections (duodenum, jejunum, ileum, and colon) after removal of content were harvested, weighed, radioactivity measured in a gamma counter (PerkinElmer 1480 Wizard or 2480 Wizard^2^), and decay-corrected tissue radioactivity concentration (kBq/g) and SUVmean were calculated.

**Lymphocyte immunophenotyping, plasma viral load, and cell-associated viral load**

Fresh blood samples collected in EDTA tubes were stained for CD3 and CD4, and analyzed by flow cytometry as previously described [2]. Plasma SIV-RNA was measured using a gag-targeted quantitative real-time/digital reverse transcription-polymerase chain reaction (RT-PCR) assay with a minimum detection threshold of 3 or 5 copies/mL as previously described [3]. Cell-associated viral load from six independent aliquots (each one run in duplicates) of SIVmac251 or SIVmac239-nef-stop viral kinetics in MT4 cells was measured and expressed as SIV-RNA copies/million cells, as previously described [4].

**Additional considerations on the reproducibility of imaging gp120 with immunoPET**

**NHP studies:**

While Kim et al.[5] do not dismiss nonspecific uptake as an explanation for the observed increase in blood pool activity (e.g. due to galunisertib-induced alteration in probe pharmacokinetics or probe-antigen plasma kinetics), they also suggest that the observed increase in heart SUV during the third cycle could be due to changes in viral antigen concentrations, consistent with the feasibility of imaging SIV *in-vivo* claimed in the previous three NHP immunoPET studies [6-8]. Unfortunately, Kim et al. [5] did not include a control group of uninfected RMs to exclude that the induced changes in SUV levels observed in their SIV-infected animals could be explained by non-specific alteration in antibody biodistribution caused by the drug regimen, hence unrelated to SIV presence. Of note, if the addition of a control group shows that, post-galunisertib administration, changes in SUV uptakes are observed only in SIV-infected animals, it may still be the result of non-specific alteration in antibody-gp120 complex kinetics, as implied by the same authors [5], hence again unrelated to gp120 molarity in tissues. Though, to our knowledge, SIV gp120 levels in whole blood have not been reported in the literature, HIV-1 and SHIV studies suggest gp120 levels of ~1 ng/mL of plasma during acute and early stages of infection [9, 10]. Assuming all gp120 in the blood is available for binding (and not occupied by endogenous anti-gp120 antibodies) and using the reported probe’s specific activity in Kim et al. [5], we estimate that <0.1% of blood pool activity may be specific binding. Moreover, we do not see evidence of the formation of probe-antigen complexes in the plasmas of any of our SIV-infected animals (both *in-vitro* and *in-vivo*) based on radio-HPLC analyses; nor do we see interference in binding to gp120 expressing cell lines from [^89^Zr]Zr-ITS103.01LS-F(ab’)_2_ incubated in plasmas of highly viremic RMs. Whether the LRA induces shedding of gp120 in the plasma to achieve levels much higher than what is expected in an infected animal matched for plasma viremia and sufficient to significantly occupy the probe is nevertheless unknown; yet, the latter would again indicate a change in biodistribution of the probe (bound to the gp120) fully non-specific, hence unrelated to changes in gp120 molarity in tissues. Again, as reported in their prior study [6], the increase in probe uptake was observed only in the axillary LN cluster but not in the spleen. It is also crucial to note that the probe uptake in larger organs (such as the liver, kidney, and spleen) after the third cycle remained notably lower compared to both radiotracers in our study. This necessitated the use of different SUV display scales ((0-1.5) in their studies [5, 6] and (0-25) in our study, suggesting that the majority of the ^64^Cu-p7D3-F(ab’)_2_ radiotracer was rapidly cleared from the body within 24 hours of intravenous infusion, in contrast with the [^89^Zr]Zr-ITS103.01LS-F(ab’)_2_ radiotracer in this study where on average ~74% remained in the body at ~48 post-injection (Fig. S4d). Notably, hepatic and renal SUV levels in our study are similar to those reported for other radiolabeled F(ab’)_2_ fragments (with ^99m^Tc, ^89^Zr, or ^64^Cu) at similar time points post-radioligand injection in rhesus macaques [11, 12] and humans (with ^111^In) [13], as well as for ITS103.01LS-F(ab’)_2_ radiolabeled with ^64^Cu (^64^Cu-DOTA-ITS103.01LS-F(ab’)_2_) and PET imaged at 24h post-injection in our program (data not shown).

**Human studies:**

As expected, the biodistributions of [^89^Zr]Zr-VRC01 (Beckford-Vera et al. [14]) and [^64^Cu]Cu-3BNC117 (McMahon et al.[15]) appeared remarkably similar. While the shorter half-life of ^64^Cu allowed McMahon et al. to image patients for up to 48h post-radiotracer injection, Beckford-Vera et al. imaged patients up to 72h post-injection. Notably, the [^89^Zr]Zr-VRC01 study saw a clear trend for higher uptake in the LNs and the gut of HIV-1 infected subjects already at Day 0 and 1, hence ruling out the longer half-life of the ^89^Zr as an explanation for the contrasting conclusions. Additionally, the affinity of [^64^Cu]Cu-3BNC117 appeared to be ~2-fold higher than that of [^89^Zr]Zr-VRC01, suggesting that the BP should be higher for the former, given that the two studies enrolled similar groups of HIV-1 infected viremic individuals.

A crucial difference in the quantification of the two clinical studies is that [^64^Cu]Cu-3BNC117 used the standardized uptake value (SUV) as an operator, similar to the monkey imaging studies, while [^89^Zr]Zr-VRC01 used SUV normalized on the aortic outflow tract (the radioactivity in the blood), i.e., tissue to blood ratio. This different measure could lead to varying interpretations of the data, especially if the blood pool activity is lower in HIV-1 infected individuals, as the published images in both papers seem to highlight, resulting in higher ratios. This issue is particularly important considering the pharmacokinetics of these two bNAbs when administered at pharmacologic doses have shown some evidence of faster clearance in HIV-1 infected individuals [16, 17], a phenomenon with an unknown mechanism but that appears to pose theoretical challenges when explained solely by sequestration due to binding to gp120 receptors. The authors of the [^89^Zr]Zr-VRC01 study do not rule out indeed the possibility that increases in relative SUV observed in LNs and the gut of HIV-1 infected individuals (both untreated and ART-treated with suppressed viral load) may indicate non-specific uptake in areas of heightened tissue inflammation with concurrent elevation in Fc receptor expression. In theory, this phenomenon, alongside other indirect effects of viral-induced pathogenesis [18], could explain the positive statistically significant associations between probe uptakes and viral dissemination levels observed in some lymphoid organs of Beckford-Vera et al. (with n=5 individuals) [14] and Samer et al. (with n=7 macaques) [6], although the latter contrasts with the lack of differences in SUV levels between infected and uninfected hosts observed in our study. Furthermore, the observation that such signal increase (attributed to an increase in viral replication) is evident in LNs but not in the spleen in the Beckford et al. study as well as in Samer et al. [6] and Kim et al. [5] studies suggest, in our view, a potential lack of robustness in the imaging systems under scrutiny because the levels of viral replication and/or gp120 (per unit volume of tissue) are known to be similar between LNs and the spleen [19, 20]. Given the partial volume effect of PET cameras, one would anticipate a higher signal in the spleen rather than in the LNs, given their size differences.


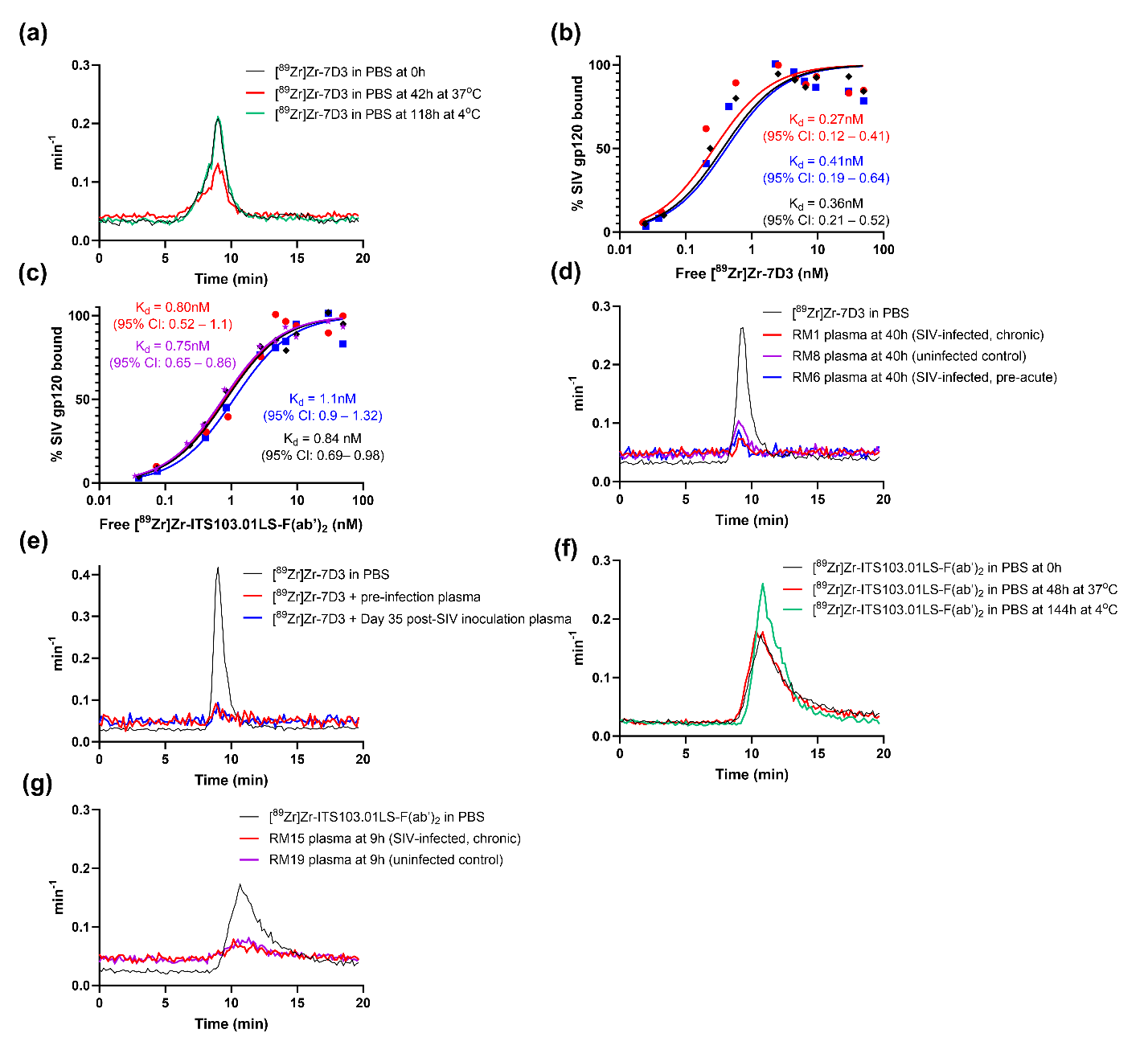


**Fig. S1** Stability of ^89^Zr radiolabeled 7D3 in PBS (black) assessed through radio-HPLC at 42h (red, radioligand stored at 37^o^C) and at 118h (green, radioligand stored at 4^o^C) post-radiolabeling (**a**). The semi-logarithmic plots of saturation binding curves of [^89^Zr]Zr-7D3 (**b**) and [^89^Zr]Zr-ITS103.01LS-F(ab’)_2_ (**c**). Representative radio-HPLC profiles of the plasmas obtained from animals at 40h post [^89^Zr]Zr-7D3 injection confirmed near 100% stability of [^89^Zr]Zr-7D3 *in-vivo* **(d)**. Radio-HPLC of the incubated [^89^Zr]Zr-7D3 and plasma at pre-infection and Day 35 post-SIVmac239-nef-stop inoculation in a representative macaque **(e)**. The stability of ^89^Zr radiolabeled ITS103.01LS-F(ab’)_2_ in PBS (black) was assessed through radio-HPLC at 48h (red, radioligand stored at 37^o^C) and at 144h (green, radioligand stored at 4^o^C) post-radiolabeling (**f**). Representative radio-HPLC profiles of the plasmas obtained from animals at 9h post [^89^Zr]Zr-ITS103.01LS-F(ab’)_2_ injection confirmed near 100% stability of [^89^Zr]Zr-ITS103.01LS-F(ab’)_2_ *in-vivo* **(g)**. All radiochromatograms were transformed into probability density curves by normalizing the area under the curve.


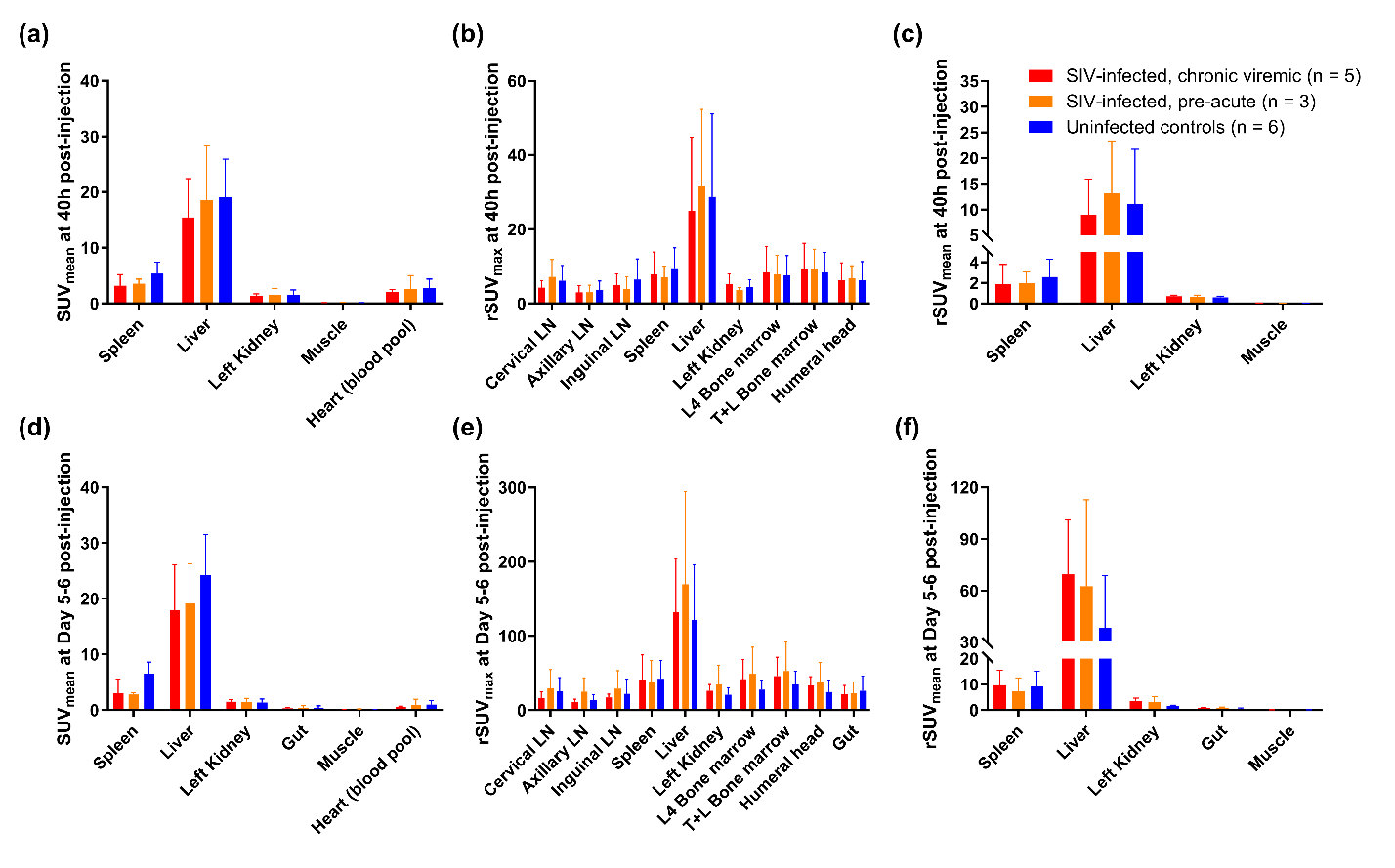


**Fig. S2** Comparison of mean (SUV_mean_), blood-adjusted maximum (rSUV_max_), and blood-adjusted mean (rSUV_mean_) standardized uptake value (SUV) in tissues among the chronically SIV-infected viremic (red), pre-acutely SIV-infected (orange), and uninfected controls (blue) at 40h (**a-c**) and Day 5-6 post [^89^Zr]Zr-7D3 injection (**d-f**). Plots are mean values and error bars are standard deviation.


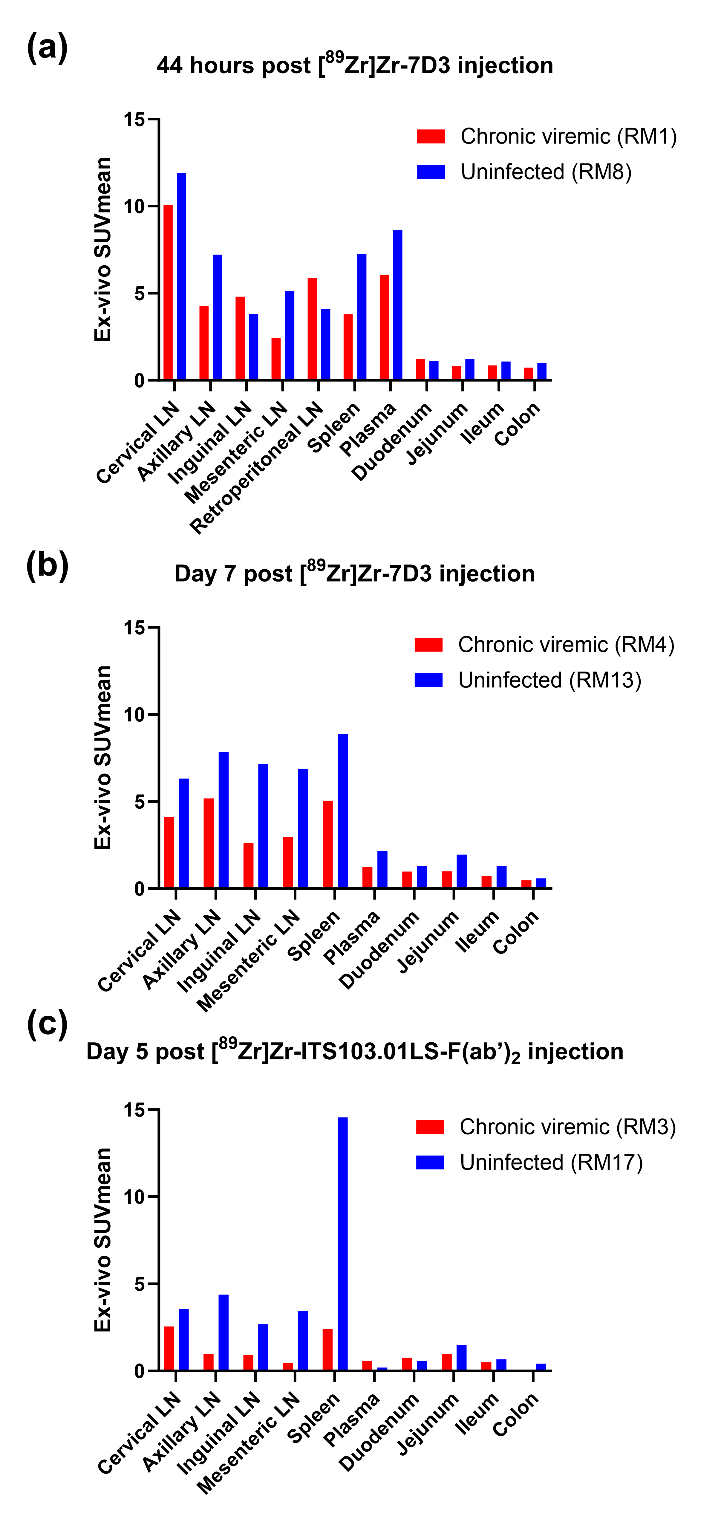


**Fig. S3** SUV in necropsy tissues obtained from one chronically SIV-infected RM (red) and one uninfected control (blue) at ~44h (**a**) and Day 7 post [^89^Zr]Zr-7D3 injection (**b**), and Day 5 post [^89^Zr]Zr-ITS103.01LS-F(ab’)_2_ injection (**c**).


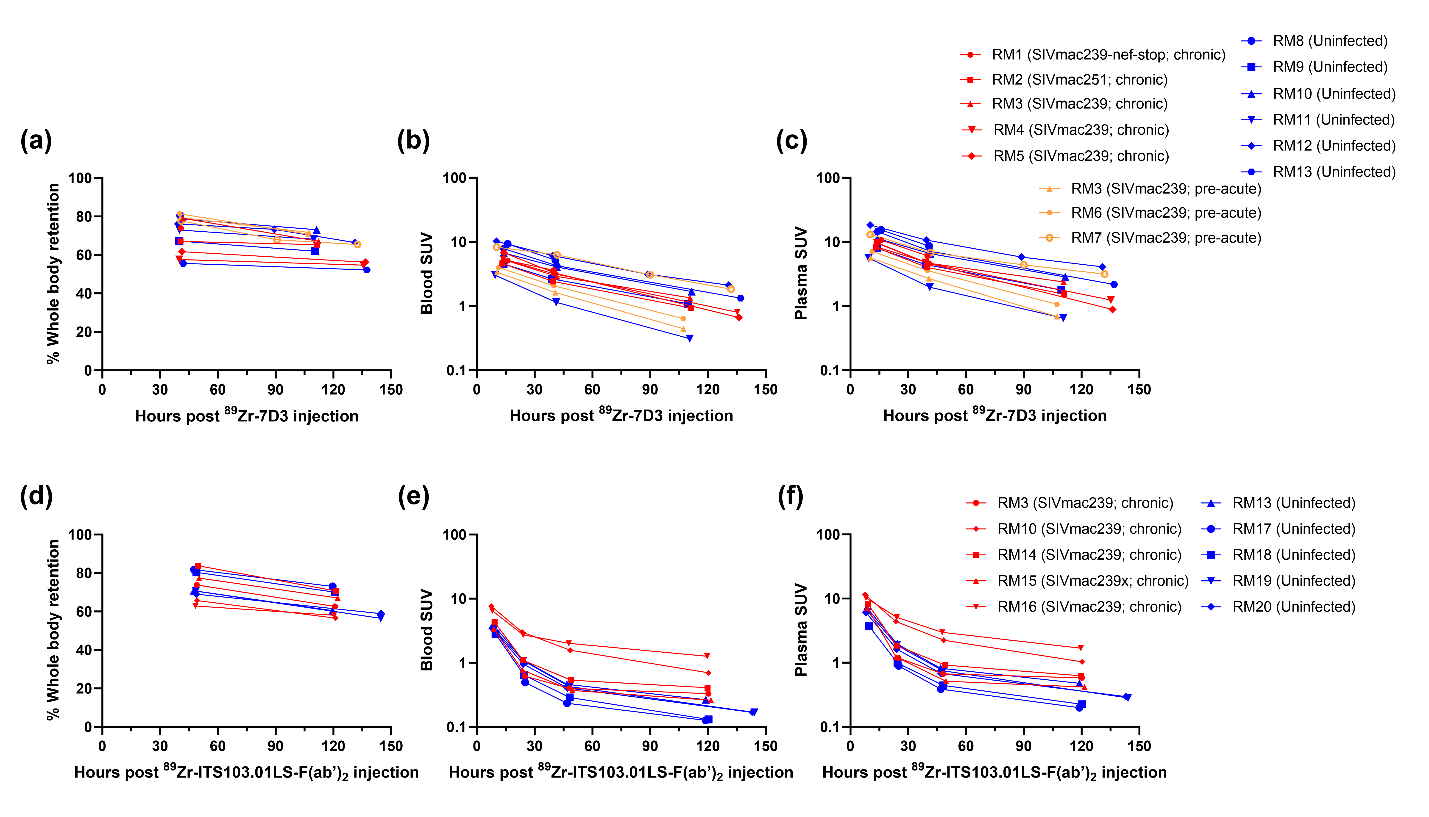


**Fig. S4** Whole-body (head to mid-thighs) clearance and standardized uptake values (SUV) in the peripheral blood and plasma of [^89^Zr]Zr-7D3 (**a, b, c**) and [^89^Zr]Zr-ITS103.01LS-F(ab’)_2_ (**d, e, f**) administered macaques.


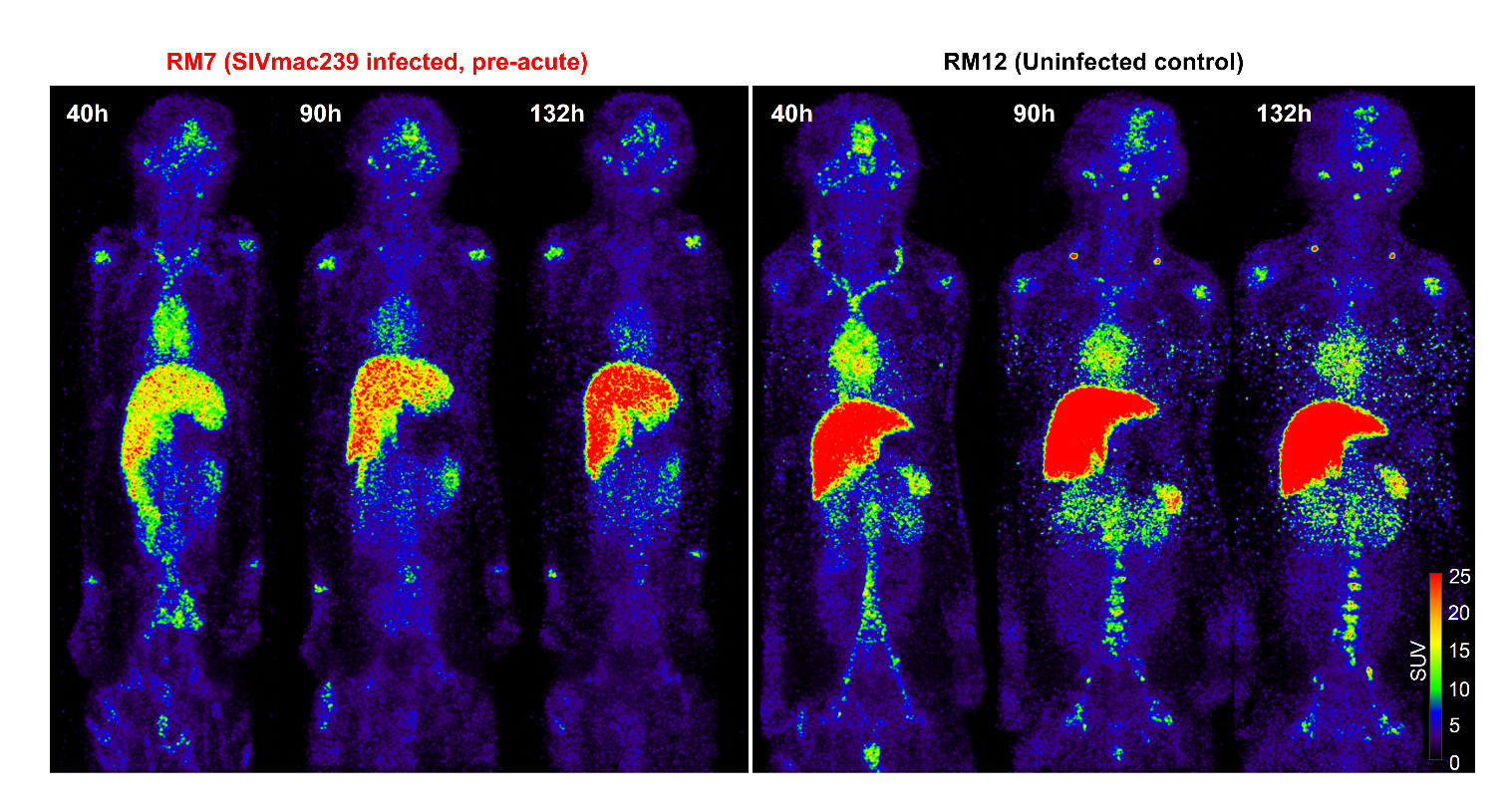
**Fig. S5** Maximum intensity projection PET images of a representative pre-acutely SIV-infected rhesus macaque (RM7) and a representative uninfected control (RM12) following administration of ~1mg mass of [^89^Zr]Zr-7D3 (~15MBq of ^89^Zr) and scanned at 40h, 90h, and Day 6 post-injection. The SIV-infected animal was inoculated intravenously with 1000 TCID50 SIVmac239, administered [^89^Zr]Zr-7D3 on Day 8 of SIV-infection, and PET/CT scanned on Day 10 (40h), Day 12 (90h), and Day 14 (132h) of infection. Tissue uptakes were displayed on the RAINBOW color scale where the red color indicates a high standardized uptake value (SUV). Both visual and semi-quantitative SUV analysis showed similar uptake in the SIV-infected RM compared to the uninfected control and the gut uptake observed at 40h had cleared at later timepoints.


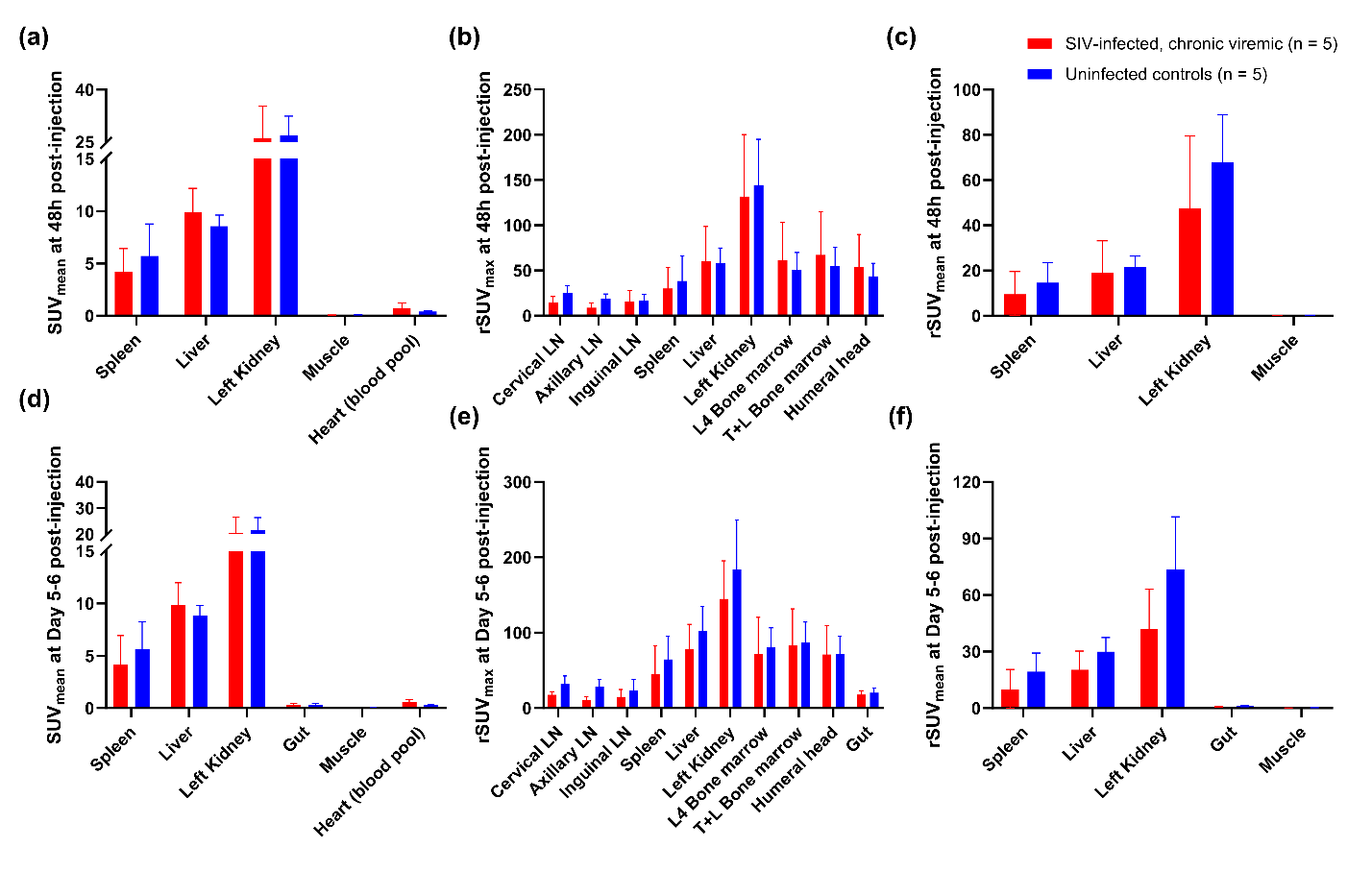


**Fig. S6** Comparison of mean (SUV_mean_), blood-adjusted maximum (rSUV_max_), and blood-adjusted mean (rSUV_mean_) standardized uptake value (SUV) in tissues among the chronically SIV-infected viremic (red) and uninfected controls (blue) at 48h (**a-c**) and Day 5-6 post [^89^Zr]Zr-ITS103.01LS-F(ab’)_2_ injection (**d-f**). Plots are mean values and error bars are standard deviation.


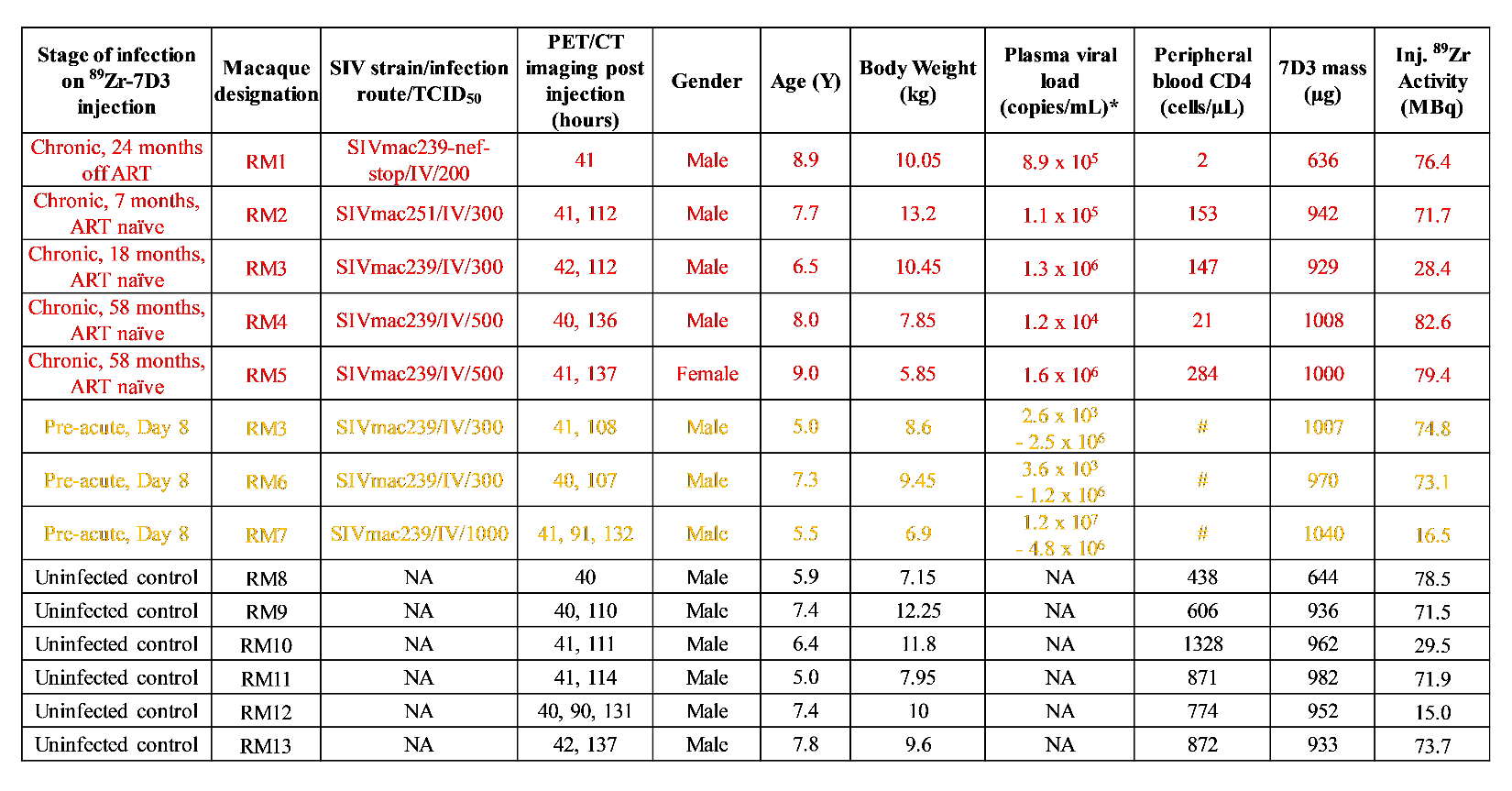
**Table S1.** Characteristics of Indian Rhesus Macaques used for *in-vivo* PET/CT imaging with [^89^Zr]Zr-7D3.

TCID_50_, median tissue culture infectious dose; IV, intravenous; ART, antiretroviral therapy; NA, not applicable

* Plasma viral load ranging from Day 7 to Day 14 post-SIV inoculation was reported for pre-acute RMs.

# Cell counts of pre-acute RMs between Day 7 and Day 14 post-SIV inoculation are not available.


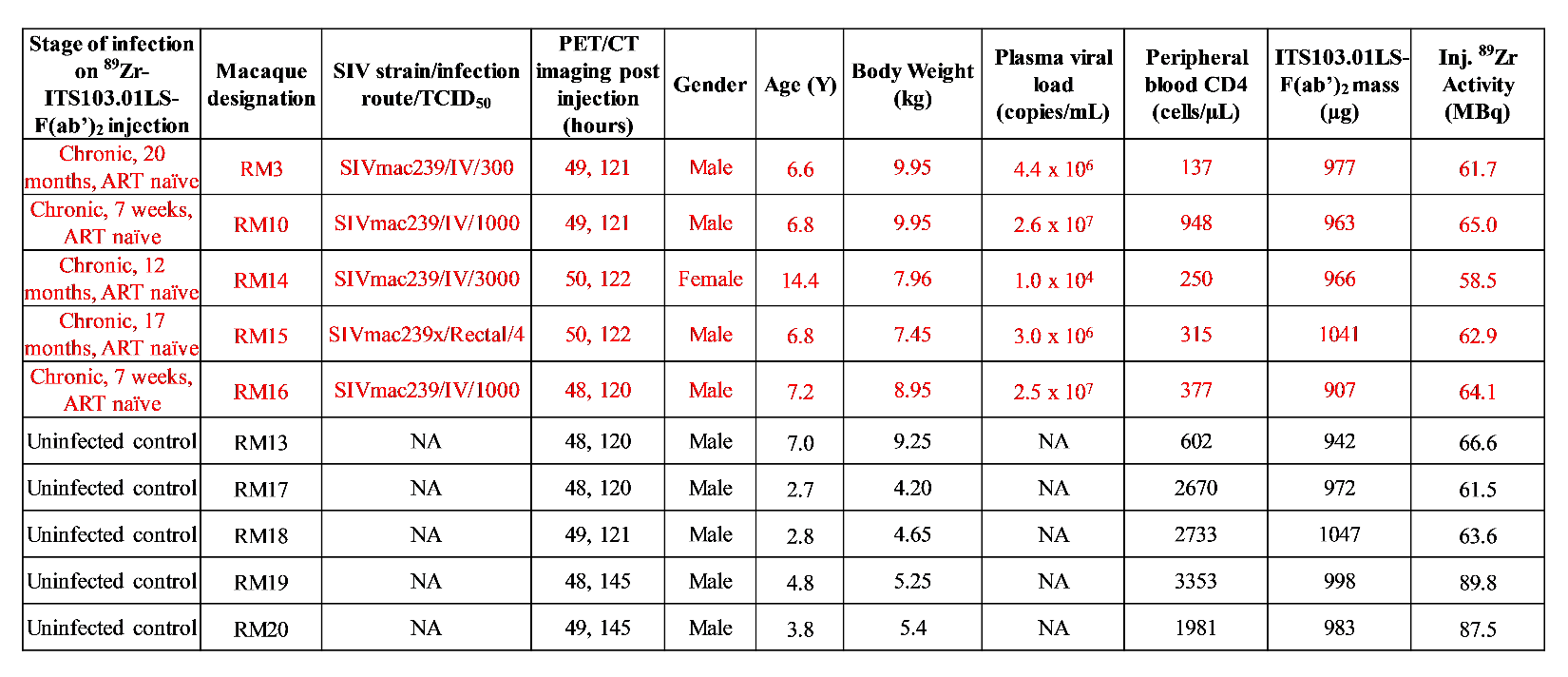
**Table S2.** Characteristics of Indian Rhesus Macaques used for *in-vivo* PET/CT imaging with [^89^Zr]Zr-ITS103.01LS-F(ab’)_2_.

TCID_50_, median tissue culture infectious dose; IV, intravenous; ART, antiretroviral therapy; NA, not applicable


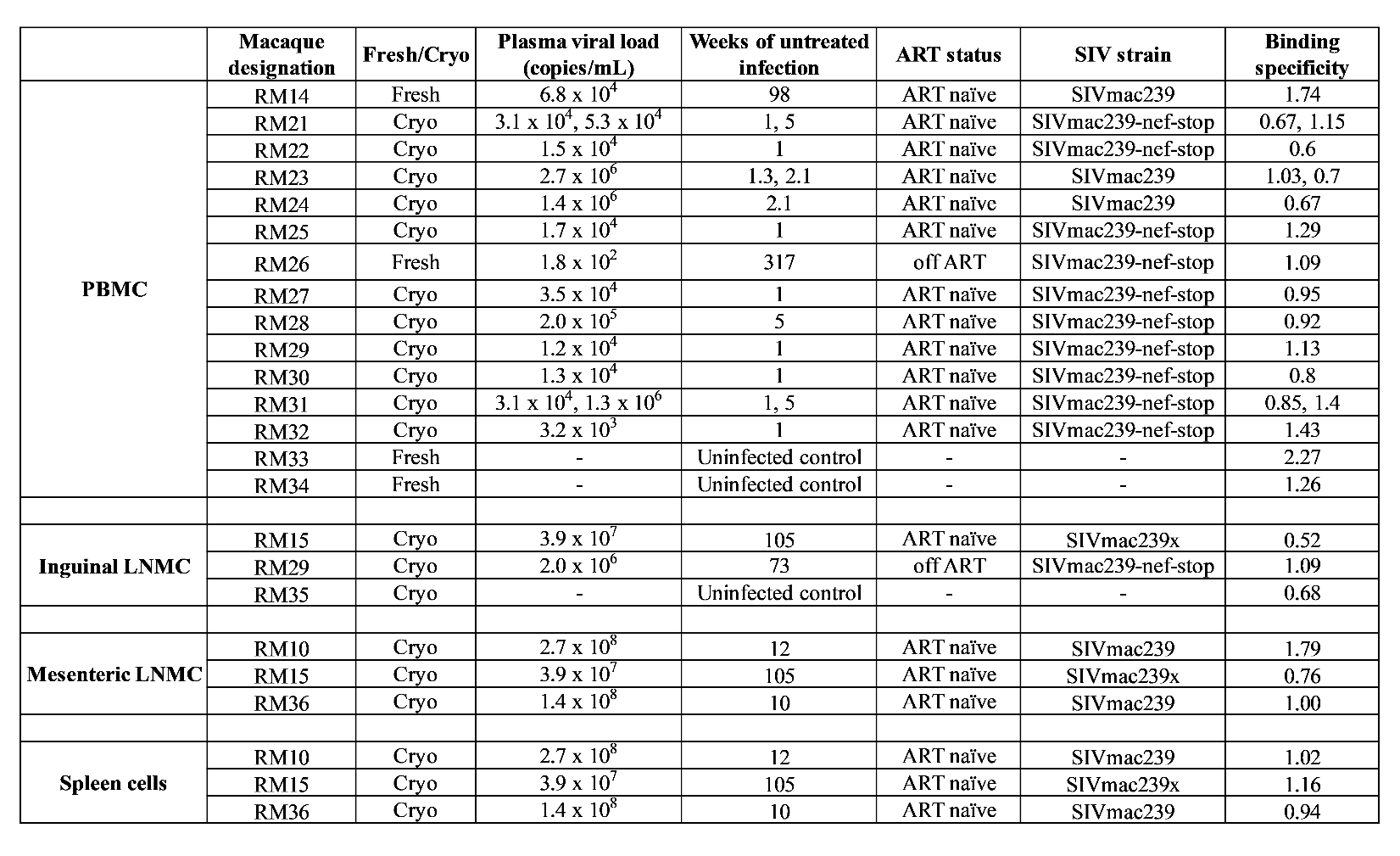
**Table S3.** Rhesus macaques used for *in-vitro* 7D3 primary cell binding studies.

PBMC, peripheral blood mononuclear cells; LNMC, lymph node mononuclear cells; ART, antiretroviral therapy


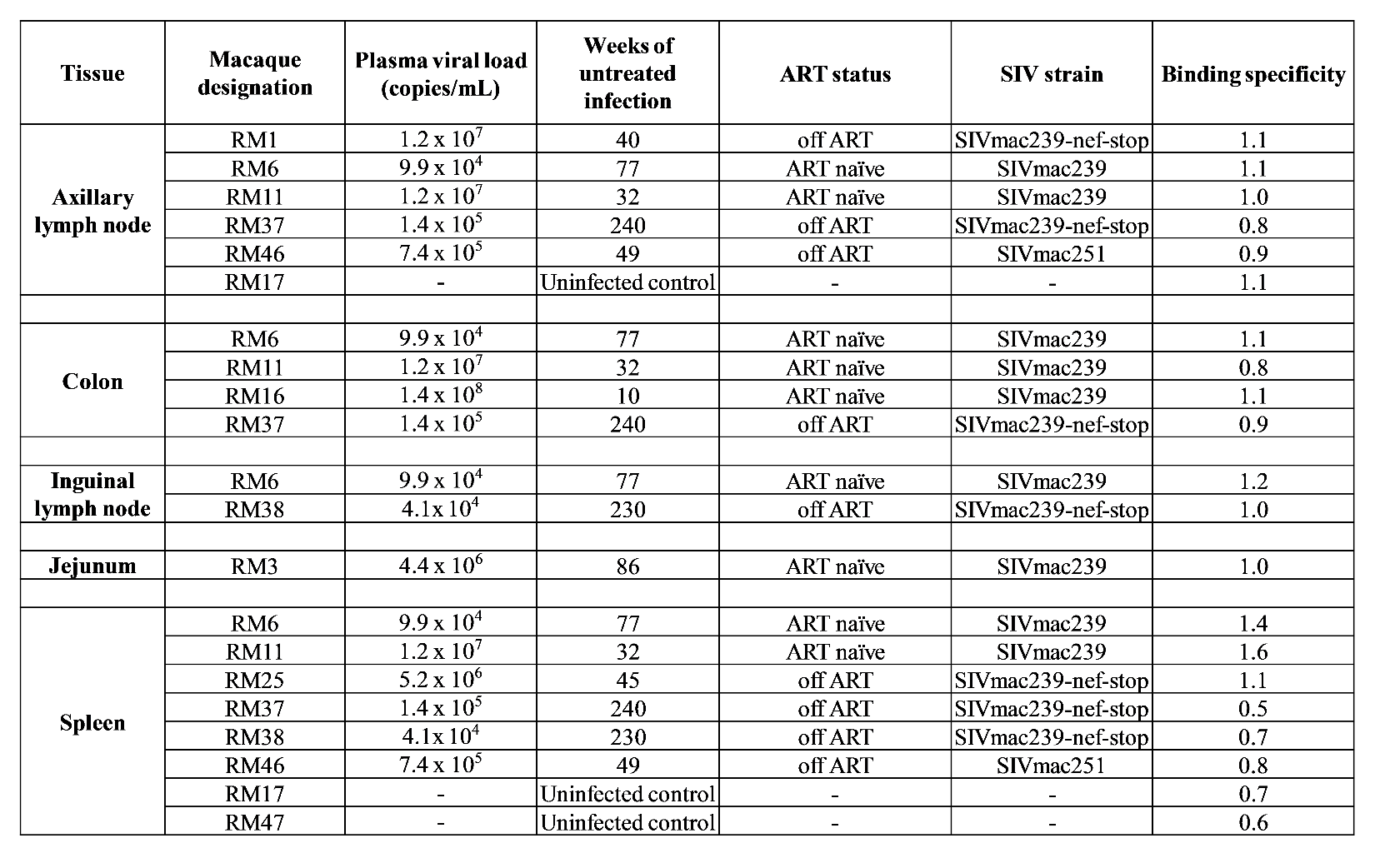
**Table S4.** Rhesus macaques used for 7D3 autoradiography studies.

ART, antiretroviral therapy


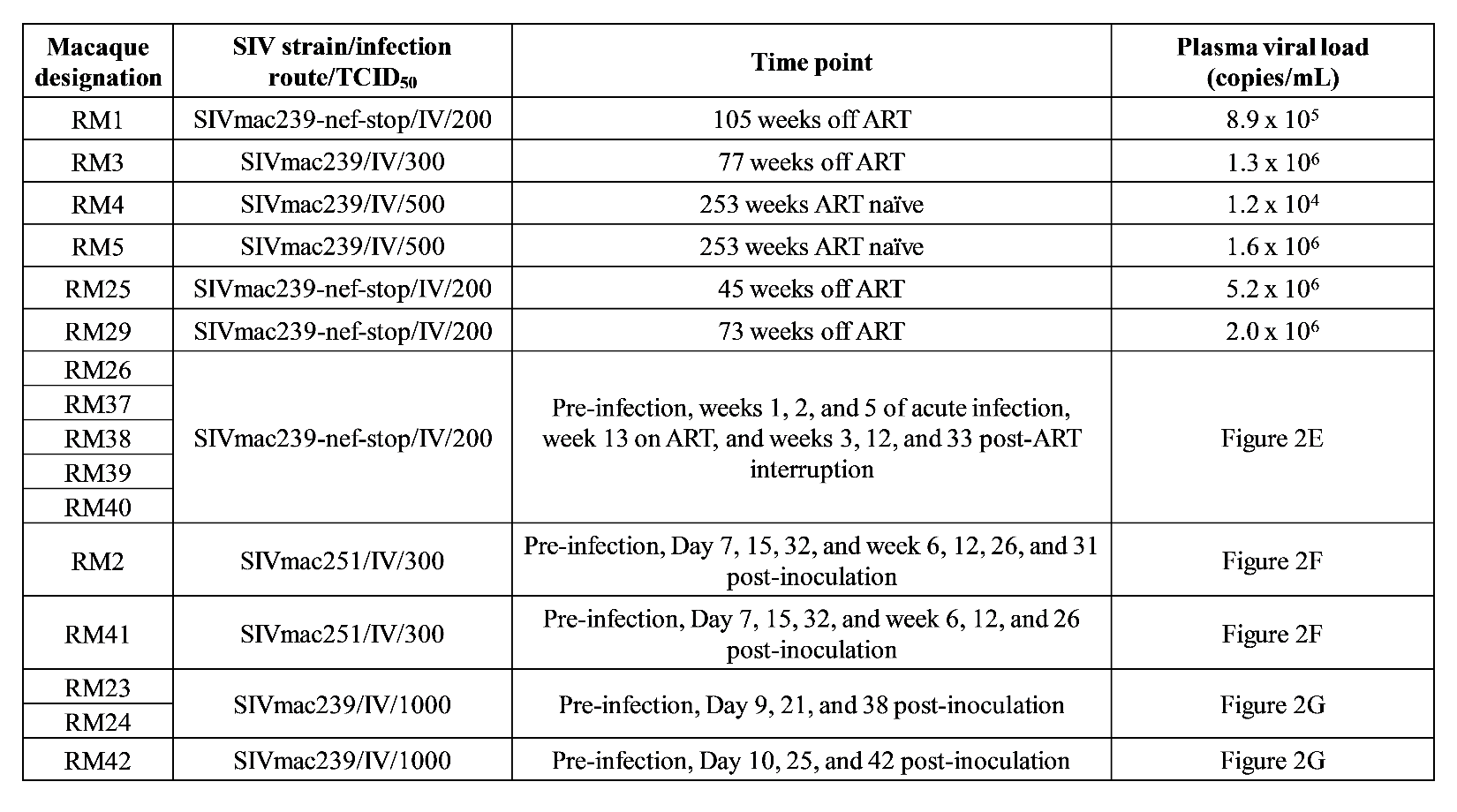
**Table S5.** SIV-infected rhesus macaques used to test the development of endogenous proteins competing for the 7D3 binding site.

TCID_50_, median tissue culture infectious dose; IV, intravenous; ART, antiretroviral therapy


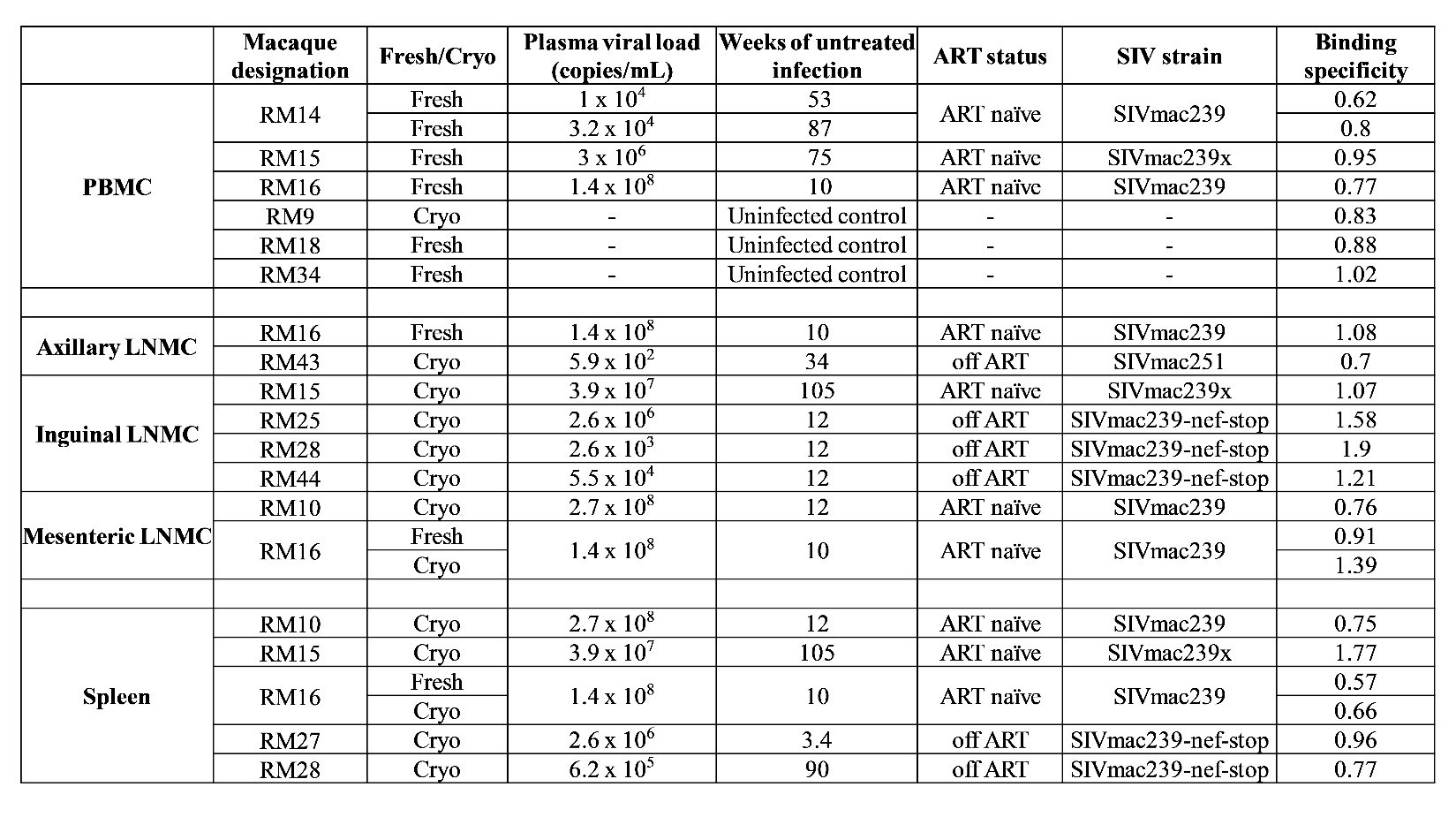
**Table S6.** Rhesus macaques used for *in-vitro* ITS103.01LS-F(ab’)_2_ primary cells binding studies.

PBMC, peripheral blood mononuclear cells; LNMC, lymph node mononuclear cells; ART, antiretroviral therapy


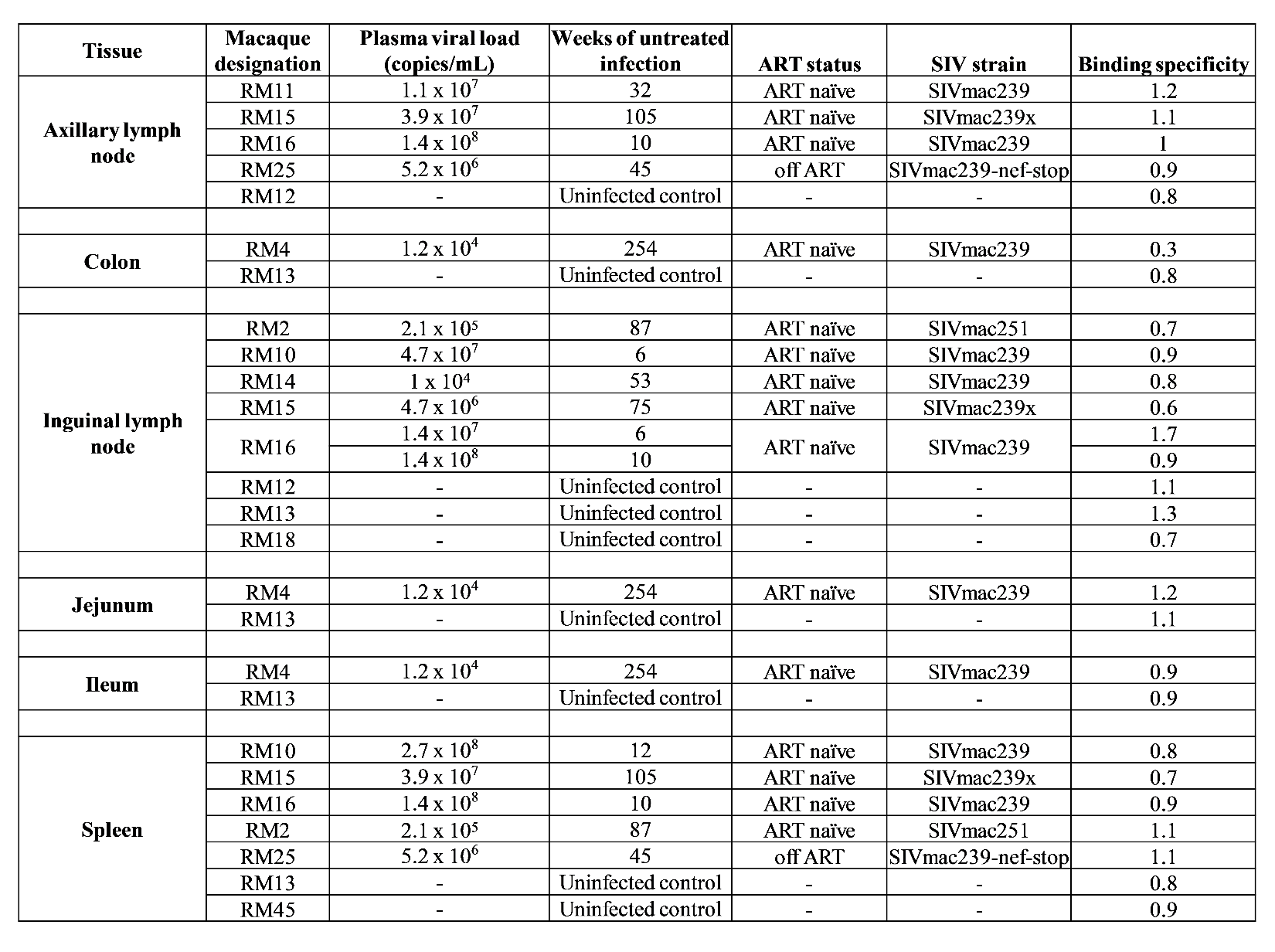
**Table S7.** SIV-infected rhesus macaques used for ITS103.01LS-F(ab’)_2_ autoradiography studies.

ART, antiretroviral therapy

**References**

1. Vosjan MJ, Perk LR, Visser GW, Budde M, Jurek P, Kiefer GE, van Dongen GA. Conjugation and radiolabeling of monoclonal antibodies with zirconium-89 for PET imaging using the bifunctional chelate p-isothiocyanatobenzyl-desferrioxamine. Nat Protoc. 2010;5:739-43. doi:10.1038/nprot.2010.13.

2. Sinharay S, Srinivasula S, Schreiber-Stainthorp W, Shah S, Degrange P, Bonvillain A, et al. Monitoring Immune Activation with Whole-Body Fluorodeoxyglucose-Positron-Emission Tomography in Simian Immunodeficiency Virus-Infected Rhesus Macaques. Immunohorizons. 2021;5:557-67. doi:10.4049/immunohorizons.2100043.

3. Cline AN, Bess JW, Piatak M, Jr., Lifson JD. Highly sensitive SIV plasma viral load assay: practical considerations, realistic performance expectations, and application to reverse engineering of vaccines for AIDS. Journal of medical primatology. 2005;34:303-12. doi:10.1111/j.1600-0684.2005.00128.x.

4. Okoye AA, Hansen SG, Vaidya M, Fukazawa Y, Park H, Duell DM, et al. Early antiretroviral therapy limits SIV reservoir establishment to delay or prevent post-treatment viral rebound. Nat Med. 2018;24:1430-40. doi:10.1038/s41591-018-0130-7.

5. Kim J, Bose D, Arainga M, Haque MR, Fennessey CM, Caddell RA, et al. TGF-beta blockade drives a transitional effector phenotype in T cells reversing SIV latency and decreasing SIV reservoirs in vivo. Nat Commun. 2024;15:1348. doi:10.1038/s41467-024-45555-x.

6. Samer S, Thomas Y, Arainga M, Carter C, Shirreff LM, Arif MS, et al. Blockade of TGF-beta signaling reactivates HIV-1/SIV reservoirs and immune responses in vivo. JCI Insight. 2022;7. doi:10.1172/jci.insight.162290.

7. Santangelo PJ, Cicala C, Byrareddy SN, Ortiz KT, Little D, Lindsay KE, et al. Early treatment of SIV+ macaques with an alpha(4)beta(7) mAb alters virus distribution and preserves CD4(+) T cells in later stages of infection. Mucosal Immunol. 2018;11:932-46. doi:10.1038/mi.2017.112.

8. Santangelo PJ, Rogers KA, Zurla C, Blanchard EL, Gumber S, Strait K, et al. Whole-body immunoPET reveals active SIV dynamics in viremic and antiretroviral therapy-treated macaques. Nature methods. 2015;12:427-32. doi:10.1038/nmeth.3320.

9. Rychert J, Strick D, Bazner S, Robinson J, Rosenberg E. Detection of HIV gp120 in plasma during early HIV infection is associated with increased proinflammatory and immunoregulatory cytokines. AIDS Res Hum Retroviruses. 2010;26:1139-45. doi:10.1089/aid.2009.0290.

10. Stevceva L, Yoon V, Carville A, Pacheco B, Santosuosso M, Korioth-Schmitz B, et al. The efficacy of T cell-mediated immune responses is reduced by the envelope protein of the chimeric HIV-1/SIV-KB9 virus in vivo. J Immunol. 2008;181:5510-21. doi:10.4049/jimmunol.181.8.5510.

11. Di Mascio M, Srinivasula S, Kim I, Duralde G, St Claire A, DeGrange P, et al. Total body CD4+ T cell dynamics in treated and untreated SIV infection revealed by in vivo imaging. JCI Insight. 2018;3. doi:10.1172/jci.insight.97880.

12. Kim I, Srinivasula S, DeGrange P, Long B, Jang H, Carrasquillo JA, et al. Quantitative PET imaging of the CD4 pool in nonhuman primates. Eur J Nucl Med Mol Imaging. 2022;50:14-26. doi:10.1007/s00259-022-05940-4.

13. Buijs WC, Massuger LF, Claessens RA, Kenemans P, Corstens FH. Dosimetric evaluation of immunoscintigraphy using indium-111-labeled monoclonal antibody fragments in patients with ovarian cancer. J Nucl Med. 1992;33:1113-20.

14. Beckford-Vera DR, Flavell RR, Seo Y, Martinez-Ortiz E, Aslam M, Thanh C, et al. First-in-human immunoPET imaging of HIV-1 infection using (89)Zr-labeled VRC01 broadly neutralizing antibody. Nat Commun. 2022;13:1219. doi:10.1038/s41467-022-28727-5.

15. McMahon JH, Zerbato JM, Lau JSY, Lange JL, Roche M, Tumpach C, et al. A clinical trial of non-invasive imaging with an anti-HIV antibody labelled with copper-64 in people living with HIV and uninfected controls. EBioMedicine. 2021;65:103252. doi:10.1016/j.ebiom.2021.103252.

16. Caskey M, Klein F, Lorenzi JC, Seaman MS, West AP, Jr., Buckley N, et al. Viraemia suppressed in HIV-1-infected humans by broadly neutralizing antibody 3BNC117. Nature. 2015;522:487-91. doi:10.1038/nature14411.

17. Lynch RM, Boritz E, Coates EE, DeZure A, Madden P, Costner P, et al. Virologic effects of broadly neutralizing antibody VRC01 administration during chronic HIV-1 infection. Sci Transl Med. 2015;7:319ra206. doi:10.1126/scitranslmed.aad5752.

18. Spadoni I, Zagato E, Bertocchi A, Paolinelli R, Hot E, Di Sabatino A, et al. A gut-vascular barrier controls the systemic dissemination of bacteria. Science. 2015;350:830-4. doi:10.1126/science.aad0135.

19. Horiike M, Iwami S, Kodama M, Sato A, Watanabe Y, Yasui M, et al. Lymph nodes harbor viral reservoirs that cause rebound of plasma viremia in SIV-infected macaques upon cessation of combined antiretroviral therapy. Virology. 2012;423:107-18. doi:10.1016/j.virol.2011.11.024.

20. Santosuosso M, Righi E, Lindstrom V, Leblanc PR, Poznansky MC. HIV-1 envelope protein gp120 is present at high concentrations in secondary lymphoid organs of individuals with chronic HIV-1 infection. J Infect Dis. 2009;200:1050-3. doi:10.1086/605695.
